# Construction and validation of a risk prediction model for delayed discharge in elderly patients with hip fracture

**DOI:** 10.1186/s12891-023-06166-7

**Published:** 2023-01-24

**Authors:** Hong Cao, Jian Yu, YaRu Chang, Yue Li, Bingqian Zhou

**Affiliations:** 1 Department of Orthopedic Trauma, Tianjin Hospital, 300211 Tianjin, China; 2 Tianjin University of Traditional Chinese Medicine, 301610 Tianjin, China

**Keywords:** Delayed discharge, Hip fracture, Risk factor, Nomogram

## Abstract

**Background:**

Because of their poor physical state, elderly hip fracture patients commonly require prolonged hospitalization, resulting in a drop in bed circulation rate and an increased financial burden. There are currently few predictive models for delayed hospital discharge for hip fractures. This research aimed to develop the optimal model for delayed hospital discharge for hip fractures in order to support clinical decision-making.

**Methods:**

This case-control research consisted of 1259 patients who were continuously hospitalized in the orthopedic unit of an acute hospital in Tianjin due to a fragility hip fracture between January and December 2021. Delayed discharge was defined as a hospital stay of more than 11 days. The prediction model was constructed through the use of a Cox proportional hazards regression model. Furthermore, the constructed prediction model was transformed into a nomogram. The model’s performance was assessed using the area under the receiver operating characteristic curve (AUC), calibration curves and decision curve analysis (DCA). the STROBE checklist was used as the reporting guideline.

**Results:**

The risk prediction model developed contained the Charlson Comorbidity Index (CCI), preoperative waiting time, anemia, hypoalbuminemia, and lower limbs arteriosclerosis. The AUC for the risk of delayed discharge was in the training set was 0.820 (95% CI,0.79 ~ 0.85) and 0.817 in the testing sets. The calibration revealed that the forecasted cumulative risk and observed probability of delayed discharge were quite similar. Using the risk prediction model, a higher net benefit was observed than when considered all patients were at high risk, demonstrating good clinical usefulness.

**Conclusion:**

Our prediction models could support policymakers in developing strategies for the optimal management of hip fracture patients, with a particular emphasis on individuals at high risk of prolonged LOS.

## Background

Of all the fractures in the elderly, hip fracture is the most serious type. Due to the characteristics of poor prognosis, multiple complications, and high mortality, hip fracture, in the elderly, is regarded as “the last fracture in life“ [1]. With the increase of age, the elderly suffer from the decline of body function, coordination, and response-ability, as well as muscle strength around the hip joint, and meanwhile, the calcium loss in the body is serious, and the bone mineral density decreases. The fracture occurs when the hip joint is slightly impacted by an external force. In recent years, with the acceleration of population aging, the increase of the elderly accordingly lead to the increase of people with hip fractures year by year. In accordance with statistics, the number of hip fractures worldwide is expected to reach 4.5 million in 2050 ( it was 1.26 million in 1990), with about half in Asia, especially in China [2]. Hip fractures account for only 14% of all osteoporotic fractures, but require 72% of the total cost, and are expected to cost more than $18.2 billion in the United States by 2025 [3]. For the patients with hip fracture, they tend to require a longer recovery and rehabilitation time, and the extension of the length of stay (LOS) may increase the incidence of long-term bedridden complications, which directly results in high medical costs, including both the individual and social medical costs [4]. Given limited medical resources and the growing elderly population, early discharge of patients with hip fractures is of particular significance. Therefore, the prediction of delayed discharge should be investigated, but no relevant assessment tools have been found. Factors reported to influence the length of hospital stay for hip fractures in older adults include osteoarthritis, women, severe postoperative pain, history of stroke, cognitive impairment, comorbidities of depression, limited postoperative weight bearing, and multiple fractures [5]. However, comorbidities, surgical outcomes, and place of discharge vary widely from patient to patient because of the differences in health care systems in different countries [6]. There is a certain degree of uncertainty, and the risk of delayed hospitalization cannot be quantified. Therefore, the independent risk factors related to the LOS of patients with hip fracture were obtained through logistics regression analysis, and a nomogram model was established, so as to better comprehend the factors affecting the LOS of patients with hip fracture, shorten the LOS, reduce the cost of hospitalization, and thus make more efficient use of medical resources. By scientifically and systematically evaluate the probability of prolonged hospitalization, the application of the nomogram model before operation can more accurately guide clinicians to intervene and treat patients in advance and reduce the occurrence of delayed discharge.

## Methods

### Study population

In total, 1305 patients who underwent treatment for hip fracture between January 2021 and December 2021 were enrolled, and the ethics committee approved the trial at our hospital. The inclusion criteria were as follows: (1) patients 65 years or older. (2) patients undergoing artificial joint replacement or intramedullary nail fixation for hip fracture. The exclusion criteria were as follows: (1) Patients who leave hospital quickly due to non-disease reasons,(2) Pathological fracture, (3) Death in hospital. Of these, 46 patients were excluded: 15 were operated with unknown type, and 31 left the hospital voluntarily.

Ultimately, 1259 patients were included in the study, and they were randomly divided into a training set (70%) and a testing set (30%). Patients in the training set were used to develop the nomogram model, whereas patients in the testing set were used to validate the resulting nomogram.

### Data collection

In this study, the factors influencing the delay of discharge were collected through the electronic medical record system. All factors were included Gender, age, hospitalization time, preoperative waiting time, operation type, injury type, fracture type, CCI, multiple injury, hypertension, coronary artery disease, diabetes, cerebrovascular disease, hemiplegia, myocardial infarction, heart failure, arrhythmia, chronic lung disease, connective tissue disease, renal insufficiency, liver insufficiency, anemia, hypoproteinemia, electrolyte disorder, malignant tumor, Alzheimer’s disease, Parkinson’s disease, arteriosclerosis of lower limbs, second fracture, delirium, digestive tract ulcer .the types of fracture and complications were diagnosed by bone physicians according to the results of imaging examination or laboratory examination after consultation. The hospitalization time is defined as the time from the day of hospitalization to the day before discharge. The 75th percentile of the patient’s hospitalization time is used as the boundary value for the extension of hospitalization time. the patients who stayed in hospital for more than 11 days were included in the delayed discharge group, while other patients were included in the normal group. If there is a missing value in the patient data, take the mean value of this variable to replace it. In addition, two team members complete the work of code entry, database establishment and statistical analysis, etc. If there are differences, a third party will solve them to ensure the accuracy of the data.

### Statistical analysis

Data were analyzed using the SPSS 22 software for Windows (IBM Corp., Armonk, NY, USA). First, the risk factors that may affect LOS were classified. Student’s t test or the Mann-Whitney U test was used to perform delayed discharge and non-delayed discharge group comparisons for quantitative variables. Categorical variables were compared using the chisquare or Fisher’s exact test. Second, all the training set factors were included in the univariate and multivariate logistic regression analyses to exclude unrelated risk factors. The independent risk factors obtained based on the multivariate regression method were used to construct a nomogram model with the “rms” package of R software (version 3.6.1). Finally, AUC, calibration curve, and DCA were used to evaluate the predictive ability and performance of the risk model. The AUC enables evaluation of the predictive accuracy and discriminative ability of nomograms. The AUC values ranged from 0.5 to 1.0, with low accuracy (< 0.5), moderate accuracy (0.5–0.7), high accuracy (0.7–0.9), and extreme accuracy (> 0.9). A calibration curve was used to compare the actual risk and predicted risk. The clinical usefulness of the nomogram was estimated by DCA based on the net benefit and threshold probabilities. Statistical tests used *p* < 0.05 as a significance level.

## Results

**Fig. 1:**
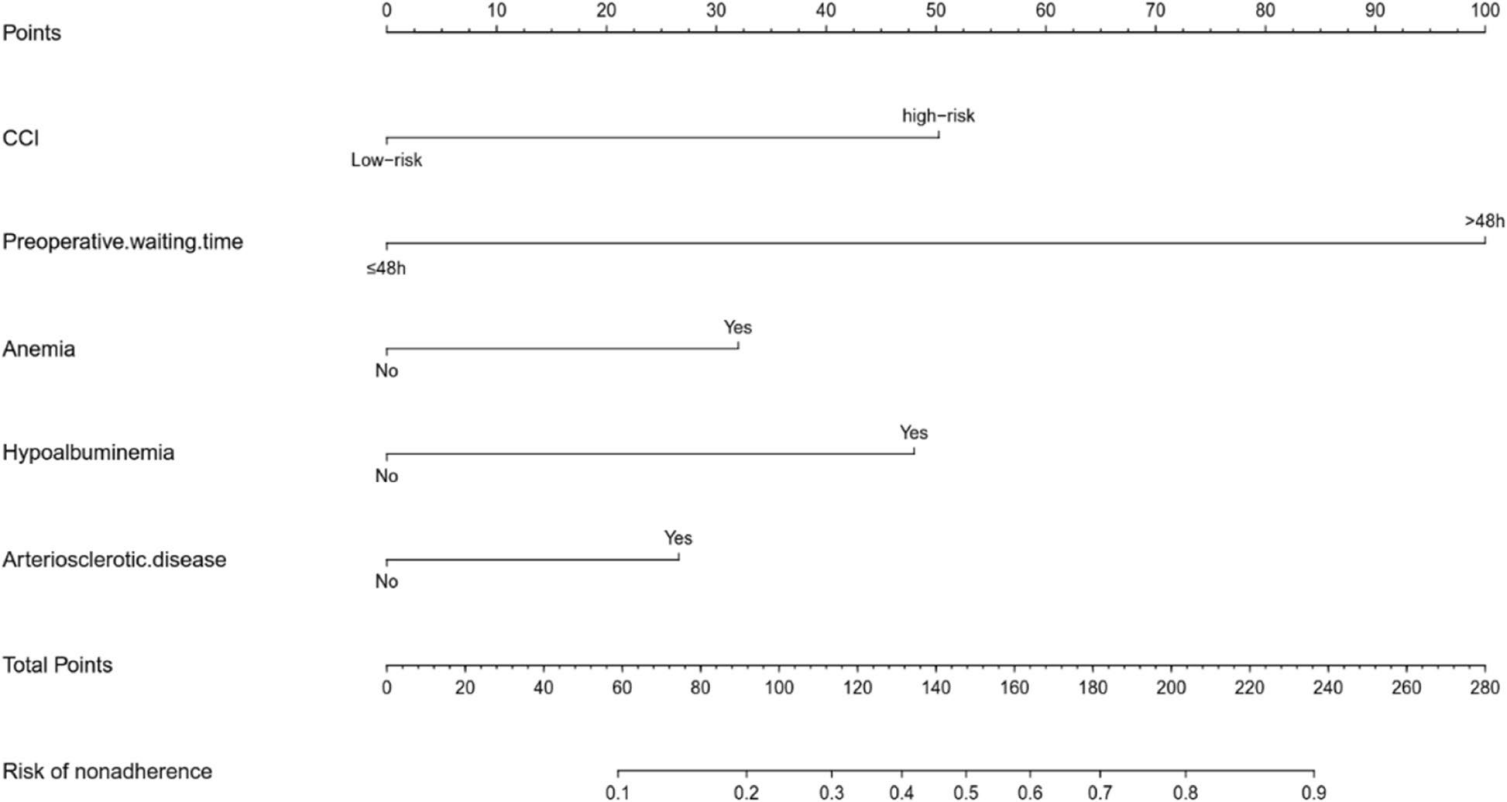
Nomogram for predicting delayed discharge in elderly patients with hip fracture

The study population comprised 1259 injured patients, with 881 patients in the training set and 378 patients in the testing set. There were 876 females (69.6%), and the average age was 76.88 years(SD: 7.26). The median LOS was 8 (6–11) days and the incidence of delayed discharge was 27.3%.In the training set, the delayed discharge group was compared with the non-delayed discharge group, and the results showed that preoperative waiting time, CCI, multiple injuries, coronary artery disease, cerebrovascular disease, hemiplegia, myocardial infarction, heart failure, arrhythmia, chronic lung disease, anemia, hypoproteinemia, electrolyte disorder, Arteriosclerosis of lower limbs, second fracture, delirium were correlated with delayed discharge. (Table 1). Risk factors (Preoperative waiting time, CCI, anemia, hypoproteinemia, Arteriosclerosis of lower limbs) for prolonged LOS were obtained by multivariate regression in the training set (Table 2). A new nomogram was constructed to evaluate the delayed discharge probability after hip fracture (Fig. 1). To apply the nomogram model, the scores of different variables are first obtained on the vertical line on the nomogram. Then, the scores of all variables are added to obtain the total score, which finally allows determination of the corresponding predicted risk value by connecting the prediction line to the total score line at the bottom of the nomogram.

**Table 1:** Demographic characteristics in the training set

Characteristics	Delayed discharge (*n* = 137)	Non-delayed discharge (*n* = 734)	*t/z/χ2*	*P value*
Age n (%)			5.657	0.059
65–80	134(56.5)	418(64.9)		
81–90	89(37.6)	201(31.2)		
> 90	14(5.9)	25(3.9)		
Preoperative waiting time n (%)			117.207	< 0.001
≤ 48 h	18(7.6)	304(47.2)		
> 48 h	219(92.4)	430(52.8)		
Gender n (%)			0.895	0.344
Female	168 (70.9)	435 (67.5)		
Male	69 (29.1)	209 (32.5)		
Type of operation n (%)			0.207	0.649
Hip arthroplasty	131(55.3)	367(57.0)		
internal fixation	106(44.7)	277(43.0)		
Injury types n (%)			1.561	0.212
Low-energy injury	225 (94.9)	623 (96.7)		
High-energy injury	12 (5.1)	21 (3.3)		
Fracture types n (%)			4.484	0.106
Femoral neck fracture	134 (56.5)	395 (61.3)		
Intertrochanteric fracture	99 (41.8)	246 (38.2)		
Subtrochanteric fracture	4 (1.7)	3 (0.5)		
CCI n (%)			82.579	< 0.001
≤ 3	170(71.7)	606(94.1)		
> 4	67(28.3)	38(5.9)		
Multiple trauma n (%)			7.596	< 0.001
No	197(83.1)	579(89.9)		
Yes	40(16.9)	65(10.1)		
Hypertension n (%)			1.920	0.166
No	102(43.0)	311(48.3)		
Yes	135(57.0)	333(57.1)		
Coronary artery disease n (%)			36.829	< 0.001
No	141(59.5)	513(79.7)		
Yes	96(40.5)	131(20.3)		
Diabetes n (%)			2.419	0.120
No	158(66.7)	464(72.0)		
Yes	79(33.3)	180(28.0)		
Cerebral vascular disease n (%)			14.561	< 0.001
No	200(84.4)	598(92.9)		
Yes	37(15.6)	46(7.1)		
Hemiplegia n (%)			13.086	< 0.001
No	192(81.0)	580(90.1)		
Yes	45(19.0)	64(9.9)		
Miocardial infarction n (%)			3.035	0.081
No	233(98.3)	642(99.7)		
Yes	4(1.7)	2(0.3)		
Heart failure n (%)			11.079	0.001
No	223(94.1)	633(98.3)		
Yes	14(5.9)	11(1.7)		
Arrhythmia n (%)			6.847	0.009
No	203(85.7)	590(91.6)		
Yes	34(14.3)	54(8.4)		
Chronic lung disease n (%)			26.625	< 0.001
No	206(86.9)	619(96.1)		
Yes	31(13.1)	25(3.9)		
Connective tissue disease n (%)			2.095	0.148
No	230(97.0)	636(98.8)		
Yes	7(3.0)	8(1.2)		
Renal insufficiency n (%)			2.283	0.131
No	229(96.6)	633(98.3)		
Yes	8(3.4)	11(1.7)		
Liver insufficiency n (%)			2.217	0.136
No	231(97.5)	638(99.1)		
Yes	6(2.5)	6(0.9)		
Anemia n (%)			114.275	< 0.001
No	150(63.3)	596(92.5)		
Yes	87(36.7)	48(7.5)		
Hypoproteinemia n (%)			115.305	< 0.001
No	127(53.6)	562(87.3)		
Yes	110(46.4)	82(12.7)		
Electrolyte disorder n (%)			93.081	< 0.001
No	165(69.6)	605(93.9)		
Yes	72(30.4)	39(6.1)		
Malignant tumor n (%)			6.520	0.011
No	12 (6.5)	14 (19.5)		
Yes	108 (113.5)	345 (339.5)		
Dementia n (%)			2.283	0.131
No	229(96.6)	633(98.3)		
Yes	8(3.4)	11(1.7)		
Parkinson's disease n (%)			0.078	0.781
No	234(98.7)	639(99.2)		
Yes	3(1.3)	5(0.8)		
Arteriosclerosis of lower limbs n (%)			66.146	< 0.001
No	170(71.7)	596(92.5)		
Yes	67(28.3)	48(7.5)		
Secondary fracture n (%)			7.190	0.007
No	231(97.5)	642(99.7)		
Yes	6(2.5)	2(0.3)		
Delirium n (%)			12.917	< 0.001
No	222(93.7)	633(98.3)		
Yes	15(6.3)	11(1.7)		

**Table 2:** Univariate and multivariate logistic regression analysis of risk factors for delayed discharge of elderly patients with hip fracture

	Univariate		Multivariate	
	OR (95% CI)	*P*	OR (95% CI)	*P*
Preoperative waiting time				
≤ 48 h	Ref		Ref	
> 48 h	10.878(6.566–18.023)	< 0.001	11.773(6.635,20.889)	< 0.001
CCI				
≤ 3	Ref		Ref	
> 4	6.285(4.077,9.689)	< 0.001	2.734(1.435,5.211)	0.002
Multiple trauma				
No	Ref		Ref	
Yes	1.809(1.182,2.769)	0.006	1.320(0.757,2.302)	1.320
Coronary artery disease				
No	Ref		Ref	
Yes	2.666(1.931,3.681)	< 0.001	1.053(0.676,1.641)	0.818
Cerebral vascular disease				
No	Ref		Ref	
Yes	2.405(1.516,3.815)	< 0.001	1.334(0.72,2.471)	0.360
Hemiplegia				
No	Ref		Ref	
Yes	2.124(1.403,3.216)	< 0.001	1.141(0.629,2.068)	0.664
Heart failure				
No	Ref		Ref	
Yes	3.613(1.616,8.075)	0.002	0.915(0.291,2.874)	0.878
Arrhythmia				
No	Ref		Ref	
Yes	1.830(1.158,2.892)	0.01	1.249(0.687,2.272)	0.466
Chronic lung disease				
No	Ref		Ref	
Yes	3.726(2.150,6.458)	< 0.001	1.082(0.504,2.322)	0.84
anemia				
No	Ref		Ref	
Yes	7.202(4.850,10.693)	< 0.001	1.920(1.045,3.529)	0.036
Hypoproteinemia				
No	Ref		Ref	
Yes	5.936(4.205,8.38)	< 0.001	2.753(1.614,4.696)	< 0.001
Electrolyte disorder				
No	Ref		Ref	
Yes	6.769(4.421,10.365)	< 0.001	1.788(0.975,3.278)	0.060
Malignant tumor				
No	Ref		Ref	
Yes	1.370(0.546,3.436)	0.503		
Arteriosclerosis of lower limbs				
No	Ref		Ref	
Yes	4.894(3.254,7.359)	< 0.001	1.775(1.026,3.071)	0.040
Secondary fracture				
No	Ref		Ref	
Yes	8.338(1.671,41.602)	0.010	1.107(0.193)	6.337
Delirium				
No	Ref		Ref	
Yes	3.888(1.760,8.592)	0.001	2.150(0.776,5.956)	0.141

**Fig. 2:**
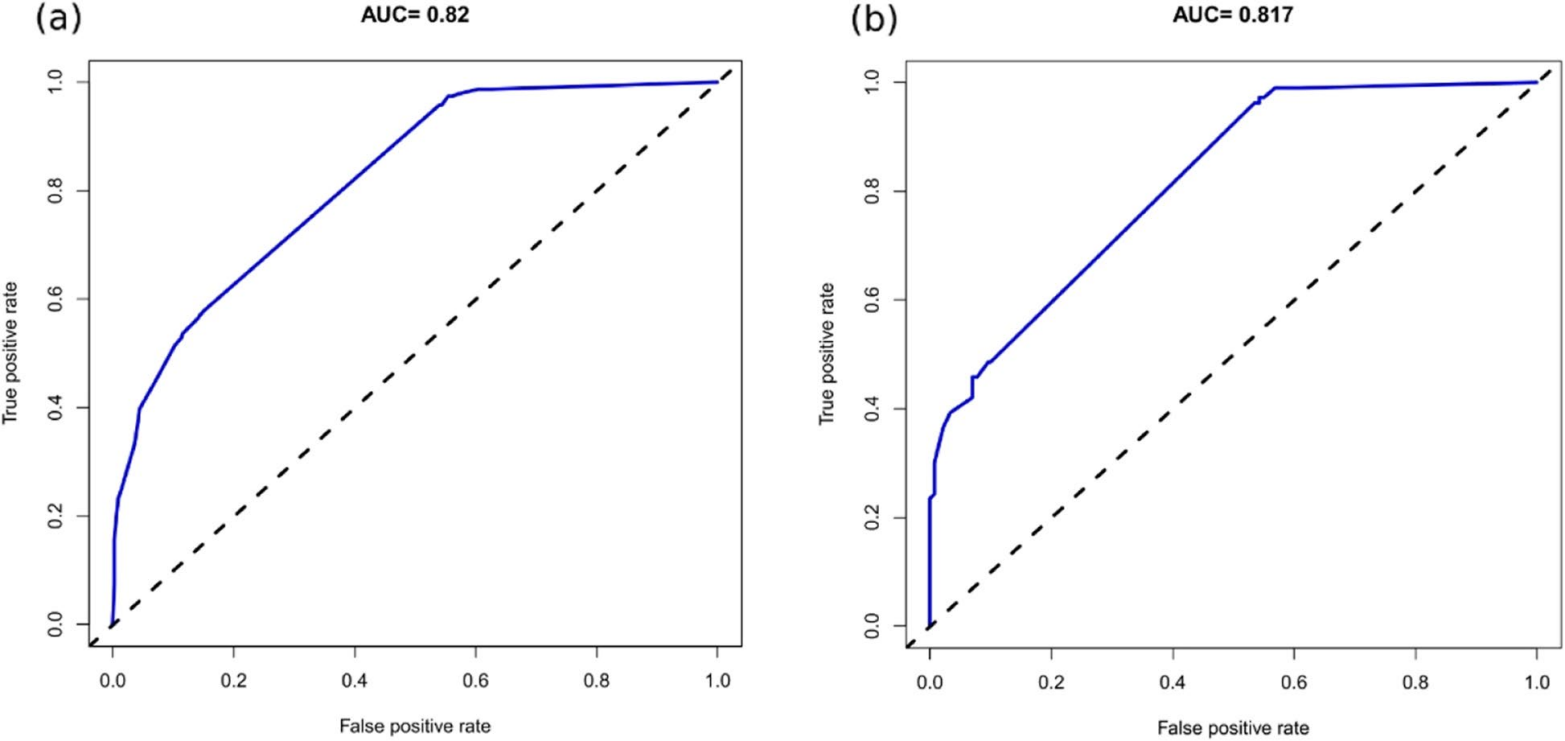
Comparison of the area under the receiver operating characteristic curve between nomogram-independent predictors in the training set (**a**) and the testing set (**b**)

**Fig. 3:**
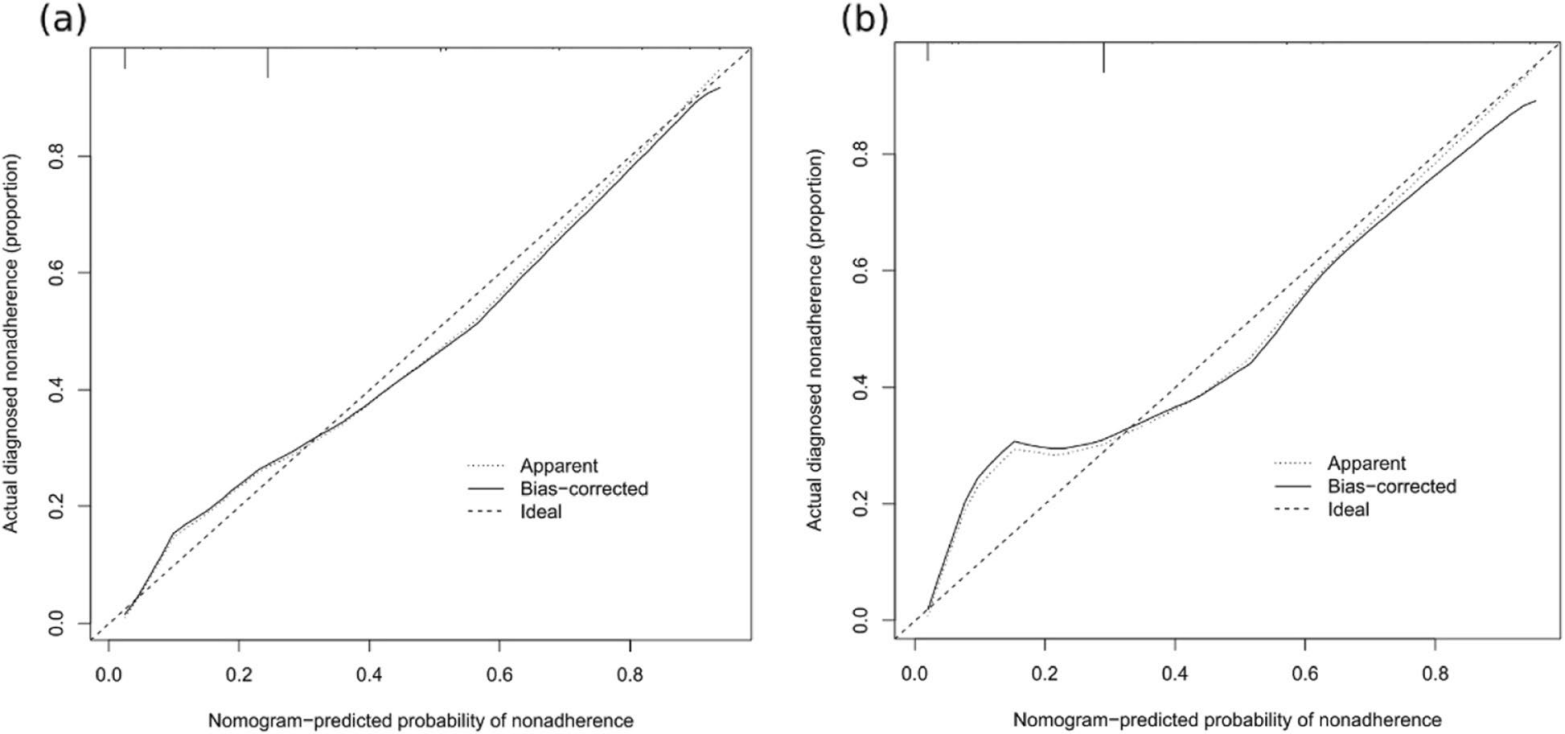
Comparison of calibration curves between the training set (**a**) and the testing set (**b**)

**Fig. 4:**
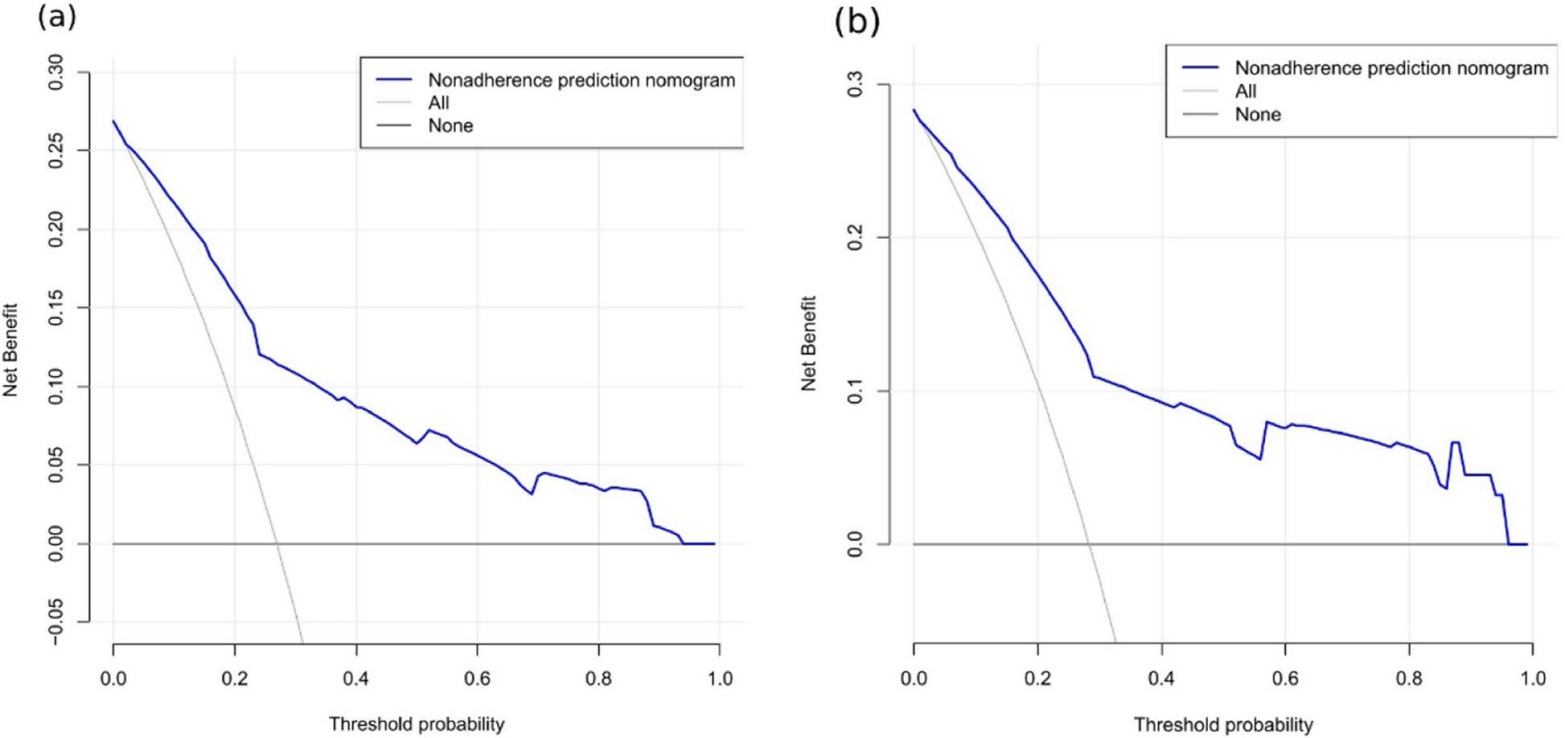
Comparison of decision curve analyses between the training set (**a**) and the testing set (**b**)

The accuracy of the nomogram model was estimated by internal validation in the training set and testing sets. This model’s c-index was 0.820, which indicated that the model was predictive with high accuracy. Furthermore, the ROC curve was constructed, and the AUC was calculated for both the training and testing sets. The AUC was 0.820(95% CI,0.793 ~ 0.847), in the training set (Fig. 2a) and 0.817 in the testing set (Fig. 2b), illustrating that the model had high discrimination. In the calibration chart (Fig. 3), the calibration curve almost coincides with the reference line in the training set and in the testing set, demonstrating good consistency between the model’s actually observed probability and the predicted probability. The DCA curves of the nomogram were shown in Fig. 4. The net benefit of using nomogram to predict delayed discharge in elderly patients with hip fracture is high when the threshold probability was between 0% and 92% in the training set (Fig. 4a) or between 2% and 93% in the validation set (Fig. 4b). Therefore, the nomogram had good clinical utility for predicting delayed discharge in elderly patients with hip fracture.

## Discussion

With the increasing aging of the population, the number of elderly patients with hip fracture increases accordingly, posing a tough challenge to health departments and orthopedic trauma surgeons. Although the surgical methods for hip fracture in elderly patients have been relatively mature, the delayed discharge of hip fracture patients still occurs frequently due to more complications and poor functional status after fracture in elderly patients. The median LOS of elderly patients with hip fracture in the present study was 10.8 days. Chen’s [7] studies have shown that patients with hip fractures have an median LOS of 12 days, indicating that discharge delays still occur in most Chinese hospitals. And studies have found that hospital stays for elderly patients with hip fractures can be reduced to four to eight days within the USA [8–10]. The differences in LOS of hip fracture patients at home and abroad may be related to the differences in discharge residence and health care system. It should be noted that the definition of LOS varies from study to study. In some studies reported, the length of hospital stay includes the acute nursing period and the early rehabilitation period. While in others, the length of hospital stay is defined as the acute period of admission, after which patients enter nursing institutions or rehabilitation institutions [11]. In addition, discharge patterns differ between hospitals and geographic regions. Most patients from private homes abroad are not discharged directly from acute wards to home, but are released to rehabilitation units [12]. In Australia and New Zealand, major trauma centers and most rehabilitation units are located in major cities. Tayhla Ryder et al. [13] found that for patients living in geriatric care institutions prior to admission, 70.8% were discharged from acute wards to geriatric care institutions and 21.2% to rehabilitation units; for patients who lived in private homes before admission, 65.8% were transferred to rehabilitation units and 18.7% went straight home. In China, due to the lack of rehabilitation units and the poor state of national economy, patients are most likely to be discharged directly to their private homes, with just a small number of wealthy patients admitted to nursing homes. Therefore, most patients in China can only complete follow-up rehabilitation in the hospital, resulting in prolonged hospital stay. In addition, for rural patients, after they return to private homes, they cannot get effective home rehabilitation services to exercise their body function, leading to a poor prognosis. As in rural hospitals, there are fewer rehabilitation beds, and rehabilitation services are dominated by a large proportion of health workers who are not rehabilitation doctors. The literature shows that long-term hospitalization can lead to adverse health outcomes in patients later in life [14, 15]. Additionally, the prolonged LOS of patients decreases the utilization rate of hospital beds and causes the waste of medical resources [16]. Therefore, the author constructed a predictive model based on logistic regression and analyzed the risk factors for prolonged LOS in patients with hip fracture, so as to take measures to maximumly avoid delayed discharge.

### Preoperative waiting time

Preoperative waiting time greater than 48 h is an influencing factor for delayed discharge of hip fracture patients. Consistent with the research results of Muhm et al. [17], the waiting time before operation has effect on the time of hospitalization. With the increase of patients’ bed time before operation, the incidence of complications will increase, such as periprosthetic infection, pneumonia, urinary tract infection, stroke, myocardial infarction, septic shock, etc., and the deterioration of nutritional status will also lead to the decline of physical function, which will eventually lead to the extension of hospitalization time [18, 19]. Sexson and Lehner et al. [20] found that early surgery can relieve pain and improve mobility, and may reduce the risk of delirium and lung infection. According to foreign studies, treatment within the first 24–72 h of fracture can achieve lower mortality and reduce postoperative complications [21]. A consensus has been reached that hip fracture patients should be operated as soon as possible, preferably within 48 h after admission [22]. Therefore, it is of great importance to give priority to a quick evaluation of hip fracture patients, and delay of operation should be avoided. At present in China, the single orthopedic mode for the treatment of elderly patients with hip fractures has a long waiting time and is of low efficiency, which can no longer meet the current clinical requirements. Therefore, it is particularly important to establish a 48-hour green channel and geriatric orthopedic ward, and adopt a multidisciplinary collaborative diagnosis and treatment mode to stabilize the physiological state of patients, avoid delayed surgery and reduce perioperative risks. For elderly patients with hip fracture, the waiting time for surgery should be shortened as far as possible in accordance with their physical conditions, and preoperative examination should be improved as soon as possible to avoid unnecessary auxiliary examination. Although patients with hip fractures can benefit from an early surgery, the surgery should also be actively optimized for patients with multiple comorbidities and poor underlying conditions.

### CCI

In this study, it was found that the hospitalization delay of hip fracture patients with CCI ≥ 8 increased by 3.326 times. The CCI scores, including the patient’s age and various complications, can reflect the overall health condition of the patient at the time of admission. Similar to the conclusions of this study, Wei [23] found that the co-disease weight of elderly patients with hip fracture (based on age-adjusted CCI assessment) was associated with the prolonged LOS. A meta-analysis also showed that there is a positive correlation between comorbid disease and the LOS of patients with hip fracture [24]. It is possible that patients with severe comorbidities may be more prone to complications and require multiple medication, so they will be given longer hospital stay for treatment and care [25, 26]. In addition, although these patients with a variety of chronic diseases are of poor self-care ability, they still need a longer period to recover to their original self-care level. Therefore, clinical attention should be focused on the occurrence of complications in hip fracture patients with a history of comorbidities. For patients with a history of chronic disease, various complications can be prevented early and preoperative health conditions can be optimized. For high-risk groups, early intervention should be taken to avoid postoperative complications and reduce the length of hospital stay.

### Anemia

Anemia is closely related to prolonged hospitalization of patients with hip fractures. Nissen Holtz et al. [27] found that anemia has a significant effect on the LOS of elderly patients with hip fracture. On the one hand, anemic patients should supplement blood volume and they need an allogeneic blood transfusion after operation, which will cause a variety of adverse reactions, affect the patient’s immune system and blood coagulation system, and increase the incidence of complications [28]. On the other hand, as the postoperative anemia in patients with hip fracture is relatively severe, the transport of nutrients in the fracture site will be affected, which weakens the body’s defense barrier and hinders the ability of self-repair, thus resulting in the invasion of pathogenic microorganisms, with the final manifestation being a rise in the incidence of complications [29]. Uncorrected anemia after hip fracture may hinder the functional recovery of patients with hip fracture and have effect on medical complications and LOS [28]. Therefore, the medical staff should evaluate the possibility of delayed discharge by reviewing the laboratory test results of patients with hip fracture and check whether they have a history of anemia before the nursing. For patients with anemia, iron supplementation can be used, and blood transfusion can also be adopted if necessary. Although blood transfusion has the risk of cross infection, low hemoglobin will lead to more complications, greatly increasing the length of hospital stay and the cost of hospitalization.

### Hypoalbuminemia

Hypoalbuminemia is associated with osteoporosis, fractures and falls. It slows down the early postoperative recovery of patients with fractures, increases the incidence of postoperative complications, prolongs the hospital stay, and causes a serious economic burden to patients’ families. Hypoalbuminemia is closely related to postoperative hematoma formation, renal insufficiency and cardiovascular adverse events. Besides, it directly impacts wound healing by affecting fibroblast proliferation and collagen synthesis [30, 31]. Moreover, hypoproteinemia will lead to the decline of patients’ immune function and the increase of the incidence of wound infection and postoperative pneumonia [32]. BOHL et al. [33] found that preoperative hypoproteinemia can significantly prolong the hospital stay and increase the postoperative readmission rate (by 40%). Similar studies have shown that preoperative hypoproteinemia can significantly increase the cost of total hip replacement and prolong the patients’ length of hospital stay [32]. In the present study, the serum albumin level of patients with hip fracture was monitored in the early postoperative stage, and it was found that patients with hypoalbuminemia and poor nutrition status should be supplemented with more nutrition, and the best nutrition supplement program should be identified.

### Arteriosclerosis of lower limbs

It was found in this study that preoperative arteriosclerosis obliterans of lower limbs were significantly related to delayed discharge of hip fracture. Patients with severe illness are featured by persistent and serious pain, ulcer and necrosis of the affected limb or toe, which may result in secondary infection and systemic toxemia. In addition, lower limb ASO will increase the prevalence of cardiovascular and cerebrovascular diseases. Studies have found that 40% of lower limb ASO patients suffer from coronary artery or cerebrovascular diseases, and 8.18% of patients suffer from three diseases at the same time [34, 35]. Therefore, the length of hospital stay of patients with hip fracture may be prolonged due to the above reasons. Antithrombotic therapy is the basic treatment of ASO, which can not only alleviate the progression of ASO, but also reduce the incidence of cardiovascular and cerebrovascular events [36, 37]. At present, low molecular weight heparin drugs are used for clinical prevention, showing sound curative effect. The combined treatment of traditional Chinese and western medicine can play the role of promoting blood circulation and Qi, removing blood stasis and relieving pain, thus increasing the tissue metabolism, so as to achieve the effect of the same treatment of specimens, and better prevent and cure thrombosis.

In this study, the construction of the prediction model for hip fracture patients with prolonged hospital stay risk was conducted. Whether the model or the internal verification of the data were of good sensitivity and specificity were investigated, and the results indicate that the prediction effect of the model is reliable and the result is stable (AUC = 0.817). The model can directly reflect the risk factors of delayed hospitalization in the formula, which can not only predict the risk of discharge delay in patients with clinical hip fracture, but also provide a basis for the follow-up medical staff to systematically evaluate the influencing factors of discharge delay. Based on this nomogram, nurses can evaluate the hospitalization time of patients with hip fracture in advance. They can speculate whether patients may have delayed discharge risk in accordance with their preoperative waiting time, CCI, anemia, hypoproteinemia, Arteriosclerosis of lower limbs and other influencing factors. It is conducive to shortening hospital stay, reducing the occurrence of postoperative complications, promoting postoperative rehabilitation, and optimizing the rational allocation of medical resources.

To shorten the LOS for elderly hip fracture patients without increasing the incidence of postoperative mortality or complications is exactly embodied by the concept of rapid rehabilitation surgery in orthopedics. Foreign scholars have confirmed that ERAS(enhanced recovery after surgery), has achieved good results in orthopedics for elderly patients with hip fracture: it can shorten the length of hospital stay for elderly patients with hip fracture, enhance community satisfaction, reduce the overall cost and improve the availability of ward beds [38–40]. However, the concept of rapid rehabilitation surgery has not been popularized in China. Over the course of the present study, although the benefits of the ERAS pathway were identified, the progress in daily practice was still slow. On the one hand, we found that the implementation of the new ERAS pathway requires multidisciplinary communication, including the emergency department, laboratory, radiology department, color ultrasound room, cardiology department, respiratory department, anesthesiology department, endocrinology department, etc., so as to rapidly improve the evaluation efficiency of admitted patients. On the other hand, it was found that the replacement of the traditional perioperative model of care progresses slowly. In order to make a difference, we put forward the following suggestions. First, it is necessary to make some changes to the internal organization of the hospital so that consensus can be reached within the hospital. In addition, the application of ERAS concept in clinical practice not only depends on orthopedic surgeons, but also on multidisciplinary cooperation, routine training and education level of doctors and nurses. Therefore, beside multi-disciplinary cooperation, rapid and effective implementation also requires nursing and other departments to develop their respective clinical pathways in advance according to ERAS pathways. Meanwhile, the concept of accelerated rehabilitation surgery is not simple optimization or blindly rote, but to develop a reasonable set of treatment plan according to the actual situation of patients. Based on the variables of this model, several measures can be taken, such as improving auxiliary examination, multidisciplinary consultation, perioperative nutrition management, early functional exercise, correction of anemia and thrombosis, etc., so as to identify a new way to solve the problem of hospitalization of hip fracture patients and optimize the quality of surgery.

### Limitations

This study is a retrospective, single-center study, some indicators are not available; the inclusion of disease heterogeneity, underlying diseases, different etiology of primary diseases, these confounding factors to establish predictive models. The effectiveness of the prediction model still needs to be further verified by multicenter, prospective studies.

## Conclusion

In this study of Chinese hip fracture patients, we used management data including clinical information to develop and vali-date risk-adjusted models to predict long-term LOS. The graph in this model shows that cci score, preoperative wait time, anemia, hypoproteinemia, and arteriosclerosis of the lower extremity are independent risk factors for prolonged hospital stay Our prediction model can support decision makers in planning strategies for the best management of hip fractures.

## Data Availability

The datasets used and/or analysed during the current study are available from the corresponding author on reasonable request.
